# Human Fecal Pollution Monitoring and Microbial Risk Assessment for Water Reuse Potential in a Coastal Industrial–Residential Mixed-Use Watershed

**DOI:** 10.3389/fmicb.2021.647602

**Published:** 2021-04-20

**Authors:** Akechai Kongprajug, Thammanitchpol Denpetkul, Natcha Chyerochana, Skorn Mongkolsuk, Kwanrawee Sirikanchana

**Affiliations:** ^1^Research Laboratory of Biotechnology, Chulabhorn Research Institute, Bangkok, Thailand; ^2^Department of Social and Environmental Medicine, Faculty of Tropical Medicine, Mahidol University, Bangkok, Thailand; ^3^Center of Excellence on Environmental Health and Toxicology (EHT), Ministry of Education, Bangkok, Thailand

**Keywords:** fecal indicator, microbial source tracking, industrial estate, land use, non-potable water reuse, freshwater, QMRA

## Abstract

Rapid economic development has caused industrial expansion into residential communities, leading to higher fecal pollution loads that could be discharged into aquatic environments. However, little is known regarding the potential microbial impact on human health. This study investigated microbial contamination from coastal industrial–residential community areas in nine sampling sites in waterways during three dry events. A general microbial source tracking (MST) marker, GenBac3, was detected in all samples from all three events, indicating continuing fecal pollution in the area, mostly from human sewage contamination. This was shown by the human-specific genetic marker crAssphage (88.9%) and human polyomavirus (HPyVs; 92.6%) detection. Enteric human adenovirus (HAdV40/41) showed three positive results only from residential sites in the first event. No spatial difference was observed for MST markers and traditional fecal indicators (total coliforms and *Escherichia coli*) in each event. Still, a significantly lower abundance of GenBac3, HPyVs, and total coliforms in the first sampling event was detected. Spearman’s rho analysis indicated a strong correlation among certain pairs of microbial parameters. Multivariate analysis revealed two clusters of samples separated by land use type (industrial vs. residential). According to factor analysis of mixed data, the land use parameter was more associated with physicochemical parameters (i.e., salinity, conductivity, water temperature, and dissolved oxygen). A Quantitative Microbial Risk Assessment (QMRA) was then conducted to estimate the annual infection risks of HAdV40/41 for non-potable water reuse purposes using predicted concentrations from crAssphage and HPyVs. The highest risks (95th percentiles) were ranked by food crop irrigation, aquaculture, and toilet flushing, at 10^–1^, 10^–2^, and 10^–3^ per person per year (pppy). Required treatment levels to achieve a 10^–4^ pppy annual infection risk were estimated. QMRA-based water treatment scenarios were suggested, including chlorination for toilet flushing reuse and depth filtration prior to chlorination for aquaculture and food crop irrigation. Microbial monitoring combined with a QMRA could provide better insights into fecal pollution patterns and the associated risks, facilitating effective water quality management and appropriate prior treatments for water reuse.

## Introduction

With rapid industrialization, the expansion of industries into peri-urban or rural communities has emerged in many geographical areas (Panyathanakun et al., 2013; Tian et al., 2017; Khan et al., 2020). The establishment of industries and industrial estates brings non-local labor, which promotes economic development in the communities. However, social, economic, and environmental components of the preceding residential communities could be transformed (Seemuang, 2018). The mismanagement of industrial pollution has also caused adverse health effects related to chemical exposure (García-Pérez et al., 2020; Khan et al., 2020; Lin et al., 2020). Efforts to reduce environmental and health impacts have been undertaken by promoting the mixed-use development concept, with careful consideration of safety to residents and environments (Altes and Tambach, 2008; Zagow, 2020). As summarized by the United Nations Industrial Development Organization (UNIDO), an eco-industrial park model is designed to consider the environmental dimension by encouraging the efficient use of resources, waste reduction and reuse, and chemical management (UNIDO, 2019). Many countries have adopted the industrial ecology concept (Piadeh et al., 2014; Industrial Estate Authority of Thailand, 2019; Susur et al., 2019; Hong and Gasparatos, 2020; Shah et al., 2020), and it was found to have successfully raised trust and improved relationship with adjacent communities (Yamsrual et al., 2019).

Although direct industrial pollutants comprise chemicals and hazardous materials, expanded populations in industrialized communities can also lead to higher fecal pollution loads that pose microbial risks to public health (Selvarajan et al., 2018). Higher total coliform counts have been reported in a river near an industrial area than in a river with no industry in both dry and wet weathers (Medeiros et al., 2017). Moreover, water quality standards for treated effluents from industries and industrial estates do not regulate bacterial parameters as microbial contamination indicators. However, some of the wastewater is from production, and some is from non-production activities, such as water use from workers (Ministry of Natural Resources and Environment, 2016; Ministry of Industry, 2017). Wastewater from toilets containing urine and fecal materials could carry waterborne pathogenic microorganisms, including protozoa, bacteria, and viruses that cause diseases in humans (Naidoo and Olaniran, 2014; Garcia-Aljaro et al., 2018). Inadequate treatment of wastewater could increase microbial contamination risks in receiving water bodies and limit their beneficial uses, such as aquaculture, recreation, and irrigation (Naidoo and Olaniran, 2014; Pandey et al., 2014). An explicit gap in the literature remains for investigations of microbial fecal pollution’s contribution and impact in receiving waters in industrialized areas.

Fecal indicator bacteria (FIB) are gastrointestinal bacteria from warm-blooded animals that have long been used as traditional fecal indicators to represent the health risks associated with pathogenic microorganisms. FIB, such as total coliforms, fecal coliforms, *Escherichia coli*, and enterococci, have been regulated in surface and coastal water quality standards worldwide (Pollution Control Department (Pcd), 1994; European Union, 2006; US EPA, 2012). Recent advancements in fecal indication incorporate microbial source tracking (MST) indicators. MST markers are also gastrointestinal microorganisms, but their presence in only specific hosts (e.g., humans, horses, pigs, and cattle) is beneficial for pollution source discrimination (Sirikanchana et al., 2014; Wangkahad et al., 2015; Ahmed et al., 2016; Garcia-Aljaro et al., 2018; Holcomb et al., 2020).

Urban and industrial development causes higher water supply demand, which can initiate conflicts among beneficiaries. Therefore, water reuse is an effective solution to resolving water scarcity for industries and residential communities (Piadeh et al., 2014). Indirect wastewater reuse, a practice referring to the utilization of surface water contaminated by either treated or untreated wastewater, has been reported for agricultural irrigation (Jeong et al., 2016; Chacón et al., 2020; Jampani et al., 2020) and recreational activities (Crank et al., 2019). However, the protection of public health is an essential aspect when evaluating water reuse and reclamation. According to the Quantitative Microbial Risk Assessment (QMRA) frameworks, enteric waterborne pathogens’ risks can be assessed. Public health risks associated with exposure to fecal microorganisms are calculated through hazard identification, exposure assessment, health effect evaluation, and risk characterization (World Health Organization, 2016). With a low abundance of waterborne pathogens, human sewage-specific MST markers have been used for QMRA analysis when the ratios of MST markers and the referenced pathogens are available (Zhang et al., 2019).

This study investigated water quality from receiving waters in industrial and residential zones to evaluate microbial impacts to public health and the water reuse potential for indirect wastewater reuse with prior treatment. The specific objectives were to (1) characterize the temporal and spatial abundance of microbial parameters (i.e., bacterial and viral fecal indicators and a pathogenic virus) in receiving waters in an industrial–residential coastal zone during base flow conditions, (2) investigate the correlations of microbial parameters with physicochemical water quality parameters and land use type (i.e., industrial and residential), and (3) assess microbial risks, estimate required treatment levels, and evaluate QMRA-based water treatment scenarios for indirect wastewater reuse. The availability of this information will assist in the prioritization of water quality and risk management strategies that can be applied to industrial communities.

## Materials and Methods

### Study Area and Sampling Locations

**FIGURE 1:**
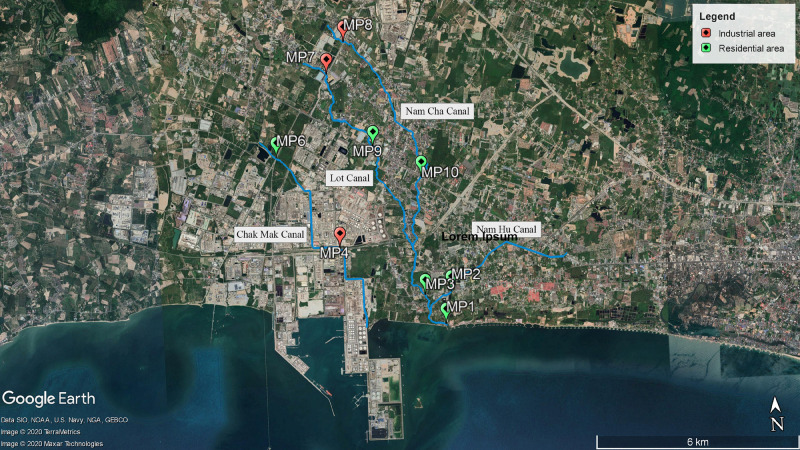
Map of sampling sites.

Sampling sites included nine locations (MP1–MP4 and MP6–MP10; MP5 was neglected after the pre-survey due to a dry canal) in four canals in the Map Ta Phut Sub-district, Rayong Province (Figure 1 and Supplementary Table 1). The Chak Mak Canal (MP4 and MP6) connects to the coast through a private land area. The Lot Canal (MP7 and MP9) combines with the Nam Cha Canal (MP8, MP10, and MP3) before joining with the Nam Hu Canal (MP2) and exits to the coast (MP1). MP1–MP3, MP6, MP9, and MP10 are situated in a residential land use type, while MP4, MP7, and MP8 are in an industrial zone (The Eastern Economic Corridor Policy Committee, 2019). Established in 1989, the Map Ta Phut industrial estate currently owns 21.997 km^2^ (Industrial Estate Authority of Thailand, 2020). This industrial park hosts seaports for transporting natural gas and goods and 57 industries comprising petrochemical, chemical and fertilizer, steel, oil, and power plants (Office of Map Ta Phut Industrial Estate, 2020). The Map Ta Phut Industrial Estate has been certified by the Industrial Estate Authority of Thailand as an eco-industrial town by matching the criteria of physical, economic, environmental, social, and managerial aspects (Industrial Estate Authority of Thailand, 2019). The industrial park coexists with residential areas and public facilities, including meeting halls, a library, and more (Industrial Estate Authority of Thailand, 2018, 2019). The industrial estate’s wastewater treatment plant has a capacity of 4,000 m^3^/day (Office of Map Ta Phut Industrial Estate, 2020). The adjacent coastal water in Pra Du Bay is a resource for fisheries and shellfish farming for residents (Seemuang, 2018).

### Sample Collection

Three sampling campaigns from the nine sampling sites were conducted in December 2019 and February and March 2020, all of which were done in the dry season to mainly represent point source pollution. As retrieved from the Hydro and Agro Informatics Institute, the precipitation records from the nearest rainwater monitoring station reported no precipitation in Events 1 and 2 and very light rain (1.5 mm) within 5 days before sampling in Event 3. The samples were collected during the low tide to minimize a tidal effect. On-site measurements of pH and water temperature were made with the YSI 60 (YSI Inc., United States), and the YSI Pro2030 instrument (YSI Inc., United States) was employed to quantify the dissolved oxygen (DO) conductivity, and salinity. Two-liter samples were collected at 30 cm below the surface for microbiological analysis (i.e., total coliforms, *E. coli*, and DNA analyses of a pathogenic virus and MST markers), according to Thailand standard (National Environment Board, 1994). Another liter of samples was grabbed at mid-depth for physicochemical parameters: total suspended solids (TSS) and biochemical oxygen demand (BOD) (National Environment Board, 1994). The samples were kept in sterile plastic bottles and placed on ice during transportation to the laboratory within 6 h. As a quality control, three field blanks and three field duplicates, each collected at each sampling campaign, were utilized.

### Physicochemical and Microbiological Water Quality Parameters

TSS were analyzed with the drying method at 103–105°C (American Public Health Association et al., 2017a), and BOD was measured using the 5-day BOD test (American Public Health Association et al., 2017b). Total coliforms and *E. coli* were simultaneously detected using a membrane filtration method with 4-Methylumbelliferyl-%-D-Galactopyranoside-Indoxyl-%-D-Glucuronide (MI medium) (US EPA, 2002).

### Water Filtration and DNA Extraction

One liter of samples (*n* = 27) was stored at 5°C for up to 3 days before a pre-acidification–filtration method, as previously described (Kongprajug et al., 2019a). Briefly, the sample pH was adjusted to 3.5 ± 0.2 using 2N hydrochloric acid before half a liter of the sample was separately filtered using a 0.45-μm-pore-size HAWP membrane (Merck Millipore, Germany). Filters were subsequently extracted with a ZymoBIOMICS DNA Microprep Kit (Zymo Research, United States), and extracts from the same samples were combined. The DNA concentration was measured with a NanoDrop 2000 Spectrophotometer (Thermo Scientific, United States). The DNA extracts were kept at −80°C until use. Three method blanks were analyzed by processing sterile laboratory water through all the steps as quality control measures.

### Quantitative PCR (qPCR) Assays

A general fecal marker, GenBac3 (Siefring et al., 2008), two human-specific fecal markers, human polyomavirus BK and JC (HPyVs) (McQuaig et al., 2009) and crAssphage (Stachler et al., 2017), and adenovirus types 40/41 (HAdV40/41) (Ko et al., 2005) were used in this study (Supplementary Table 2). The abovementioned MST markers were selected because they have been investigated and validated in Thailand (Kongprajug et al., 2019a, 2020; Sangkaew et al., 2021), and HAdV40/41 was chosen because it has been identified as the second most prevalent following the subgroup C from child patients with acute gastroenteritis in Thailand (Kumthip et al., 2019). A 20-μl qPCR reaction contained 0.8 μl of each 10 μM forward and reverse primers, 0.4 μl of 10 μM probe, 2 μl of extracted DNA, 6 μl of 1 μg/μl BSA, and 10 μl of the 2X iTaq Universal Probes Supermix (Bio-Rad, United States). The qPCR cycling conditions comprised an initial denaturation at 95°C for 3 min followed by 40 cycles of a denaturation step at 95°C for 20 s and a combined annealing and elongation step for 1 min at 55°C for HPyVs and HAdV40/41 and at 60°C for GenBac3 and crAssphage. The qPCR reactions were performed with the QuantStudio^TM^ 3 Real-Time PCR System (Applied Biosystems, Thermo Fisher Scientific, United States), and the results were examined using the QuantStudio^TM^ Design and Analysis Software with an automatic baseline and manual adjustment of the threshold values for GenBac3 (0.150), crAssphage (0.036), HPyVs (0.025), and HAdV40/41 (0.020). Environmental samples were run in duplicate, and the averaged *C*_*q*_ was used to calculate gene copy numbers when the standard deviation of *C*_*q*_ was less than 0.5; otherwise, an additional run was undertaken. The qPCR protocol was conducted according to the Minimum Information for Publication of Quantitative Real-Time PCR Experiments (MIQE) guidelines (Bustin et al., 2009). For each instrumental run containing environmental samples, the DNA standard, at a concentration of 5 × 10^4^–5 × 10^5^ copies/reaction, was run in triplicate as a calibration control according to a mixed model (Sivaganesan et al., 2010; Kongprajug et al., 2020). No-template controls (NTCs) in triplicate were also included in every qPCR instrumental run.

### Standard Curves, Limits, and Inhibition Analysis

Standard curves were constructed using synthetic plasmid standards (Invitrogen, Thermo Fisher Scientific, United States) for crAssphage (Kongprajug et al., 2019b), and synthetic linear DNA fragments were used for GenBac3 (string 1) (Kongprajug et al., 2019a) and for HPyVs and HAdV40/41 (string 2) (Supplementary Figure 1). The standard curves were obtained from four replicates of individual instrumental runs according to the mixed model method, each with a triplicate of six 10-fold concentrations, ranging from 5 × 10^1^ to 5 × 10^6^ copies/reaction. The assay limit of detection (ALOD) was the lowest concentration in copies/reactions that showed positive detection in all 10 standard replicates. The assay limit of quantification (ALOQ) was considered to be the lowest concentration in copies/reactions of the target gene that could be correctly quantified—in this case, the lowest concentration in the standard curve with a standard deviation of *C*_*q*_ of less than 0.5 (Haugland et al., 2010; Kongprajug et al., 2020). The method limit of quantification (MLOQ) was calculated for each sample as copies/100 ml by incorporating the sample’s filtration volume and DNA extracted volume. An inhibition analysis was performed with the dilution method using three dilutions (0.5, 1.0, and 2.0 μl) of the DNA templates in duplicates. The GenBac3 assay was administered, and *C*_*q*_ values for each dilution were plotted against the DNA concentration, and an *R*^2^ of <0.90 suggested significant inhibition. Three field blanks and three method blanks were also investigated with HPyV and crAssphage assays (Sangkaew et al., 2021).

### Statistical Analyses

A total of 13 water quality parameters for 27 samples were analyzed in R (R Core Team, 2019). Normality was assessed using the Shapiro–Wilk test. The two groups’ significant differences were discerned using a *t*-test for normal data and the Mann–Whitney test for non-normal data. Significant differences for more than two groups were tested using one-way analysis of variance (ANOVA), with Tukey’s multiple comparisons for normal data and Kruskal–Wallis test with Dunn’s multiple comparisons for non-normal data. Significant difference tests for paired samples were carried out with a paired *t*-test for normal data sets and the Wilcoxon signed-rank test for non-normal data.

The data sets containing data below the MLOQ, so-called non-detects, were analyzed via non-parametric survival analyses (Helsel, 2012). The data sets’ summary statistics, including non-detects, were calculated with the non-parametric Kaplan–Meier method, with Efron bias correction. The significance of the differences for these data sets was examined via the generalized Wilcoxon test (Peto–Prentice test) for multiple comparisons, with Holm’s bias correction. A paired sample comparison was also made with the paired Prentice–Wilcoxon test. Correlation analysis among multiple parameters was performed using Spearman’s rho on *U*-Score rank.

To study the interaction between the pollution sources and anthropogenic activities, the types of land use (i.e., industrial and residential) were analyzed with fecal markers and water quality parameters using factor analysis of mixed data (FAMD), which is a type of principal component analysis (PCA) for examining a data set containing both quantitative and qualitative variables (Pages, 2004).

### Risk Assessment and Sensitivity Analysis

A risk assessment following the QMRA framework comprises four basic steps: hazard identification, exposure assessment, dose–response model, and risk characterization (World Health Organization, 2016). A static QMRA was administered to evaluate infection risks from the HAdV40/41 pathogenic virus. First, in the hazard identification step, crAssphage and HPyVs were selected to estimate concentrations of HAdV40/41 pathogen using their detectable ratios in the receiving water. HAdV40/41 was the target pathogen for health risk assessment owing to the occurrence of waterborne illness, abundant in wastewater, causing gastrointestinal (GI) illness (Fong et al., 2010) and listed in the US EPA Contaminant Candidate List 4 (CCL4) (US EPA, 2016). The probability density functions (PDFs) of HAdV40/41, crAssphage, and HPyVs were modeled as a log-normal distribution from the measured concentrations. Then, the distribution ratios of HAdV40/41:crAssphage and HAdV40/41:HPyVs were used to simulate HAdV40/41 concentrations using the Monte Carlo approach at 10,000 iterations. The viable ratio of HAdV40/41 was assumed as 0.001 (Crank et al., 2019). In the second step, an exposure assessment was performed by considering three exposure scenarios: toilet flushing, aquaculture, and food crop irrigation, according to current practices in Map Ta Phut communities (Seemuang, 2018). Exposure factors, including exposure type, exposed water volume, and exposure frequency for three water reuse scenarios and a natural decay effect, are listed in Supplementary Table 3. A uniform distribution was considered for the water volume and the yearly frequency, and a triangular distribution for the natural decay was used, as previously reported (Chhipi-Shrestha et al., 2017). In the third step, the adenovirus dose–response relationship has been established and described by an exponential model for calculating the probability of daily infection (*P*_*inf*_) as 1 − exp(−0.4172*d*), where *d* represents the dose of viable pathogens (Chigor et al., 2014; Katukiza et al., 2014). Lastly, in the risk characterization step, the probability risk of infection was estimated by integrating hazard identification, exposure assessment, and dose–response model to characterize the risk. Risk characterization involves the determination of a health outcome with the risk of infection. The annual risk of infection (*P*_*y*_) was calculated with the following equation: 1 − (1 − *P*_*inf*_)^*f*, where *f* is the frequency of exposure per year. The Monte Carlo approach for simulation was run for 10,000 iterations for each scenario. The mean, median, 5th, 25th, 75th, and 95th percentiles of *P*_*y*_ were calculated using Oracle Crystal Ball v.11.1.2.4.850 software. Additional data visualization was completed in KaleidaGraph version 4.5.4. The estimated *P*_*y*_ was compared to the US EPA annual infection risk benchmark for finished drinking water of no more than 1 case per 10,000 persons per year (10^–4^ pppy) (US EPA, 1989). The required log_10_ reductions of HAdV40/41 were calculated for each water reuse scenario to achieve the US EPA risk benchmark. The recommended treatment technologies were evaluated using log-removal information, as reported previously (Supplementary Table 4).

Moreover, each input variable’s effects to risk calculation were assessed using a sensitivity analysis with Oracle Crystal Ball software. Each parameter’s significance was characterized by its correlation coefficient values with the probability risks, where a higher value indicated a more significant contribution of risks. Contribution to variance was calculated by squaring the rank correlation coefficient values and normalizing to 100%.

## Results

### The qPCR Standard Curve Characteristics, Limits, and Controls

Standard curves for GenBac3, HPyVs, crAssphage, and HAdV40/41 were characterized, with PCR efficiencies ranging from 90.01 to 101.91% (Supplementary Table 5). The ALOD ranged from 20 copies/reaction (GenBac3 and crAssphage) to 40 copies/reaction (HPyV), while the ALOQ was 50 copies/reaction for all assays. The MLOQ in the negative samples were measured as 2.10–2.40, 2.40–3.00, and 2.40 log_10_ copies/100 ml for HPyVs (*n* = 2), crAssphage (*n* = 3), and HAdV40/41 (*n* = 24), respectively. Inhibition was detected in 14.8% of the samples (*n* = 4), each using a 0.5-μl DNA template to alleviate the inhibition effect. Laboratory reproducibility was examined when all four qPCR assays were analyzed in field duplicates, showing acceptable coefficients of variation in three representative samples (0.37–8.10%; Supplementary Table 6). NTCs were found to be negative with crAssphage (*n* = 12) and HPyV (*n* = 21). Two of 15 and 25 of 30 NTCs were positive for HAdV40/41 and GenBac3, respectively. However, their abundance of 1.83–2.02 copies/reaction and 1.30–1.41 copies/reaction was very low compared to those of the samples, demonstrating negligible effects. Furthermore, all the field and method blanks were negative, showing no contamination in the field and laboratory processing steps.

### Physicochemical Water Quality Parameters

Seven physicochemical parameters were measured for nine sampling sites during three sampling events (*n* = 27) (Table 1). TSS was negative (<2.5 mg/l) in one sample, while the rest ranged from 3.0 to 307.0 mg/l. BOD ranged from 2.5 to 72.0 mg/l, DO from 3.5 to 11.7 mg/l, conductivity from 0.159 to 24.700 mS/cm, salinity from 0.2 to 8.9 ppt, pH from 5.93 to 9.14, and water temperature from 28.3 to 35.4°C. No spatial distributions, except conductivity and salinity, were significantly different among all the sampling sites (*p* < 0.05; one-way ANOVA with Tukey’s multiple comparison test), with the highest values in MP1 and MP2 near the exits to the coast (Supplementary Table 7). For each sampling site, no temporal change was found for conductivity and salinity (*p* > 0.05; Wilcoxon signed-rank test) and temperature (*p* > 0.05; paired *t*-test) (Supplementary Table 8). The pH was higher in Event 1 than in the other two events, while BOD was significantly lower in Event 1 than in Event 3 (*p* < 0.05; Wilcoxon signed-rank test). DO was found to be higher in Event 1 than in Event 3 (*p* < 0.05; paired *t*-test), and TSS were higher in Event 3 than in Event 2 (*p* < 0.05; paired Prentice–Wilcoxon test).

**TABLE 1:** Descriptive statistics of microbial and physicochemical water quality parameters.

Parameter	Normality^*a*^	Land use	Number of samples	Number of non-detects	Min^*b*^	25th percentile^*b*^	Median^*b*^	75th percentile^*b*^	Max^*b*^
GenBac3 (log_10_	0.0956	Industrial	9	0	4.89	5.41	5.91	7.81	7.90
copies/100 ml)	0.7987	Residential	18	0	4.68	5.95	6.85	7.25	8.24
HPyVs (log_10_	0.8151	Industrial	9	1	<2.40^*c*^	3.08	3.36	4.09	5.10
copies/100 ml)	0.9509	Residential	18	1	<2.10	3.04	3.64	4.41	5.07
crAssphage (log_10_	0.7540	Industrial	9	2	<3.00	3.11	3.82	4.44	4.96
copies/100 ml)	0.3037	Residential	18	1	<2.40	3.89	4.32	4.56	5.21
HAdV40/41 (log_10_	0.9669	Industrial	9	9	<2.40	<2.40	<2.40	<2.40	<2.40
copies/100 ml)	0.3037	Residential	18	15	<2.40	<2.40	<2.40	<2.40	3.87
Total coliforms	0.0013	Industrial	9	0	453	11,400	43,667	985,000	2,253,333
(CFU/100 ml)	<0.0001	Residential	18	0	11,200	50,167	72,167	829,167	7,333,333
*E. coli* (CFU/100 ml)	0.0002	Industrial	9	0	50	268	4650	701,667	1,713,333
	<0.0001	Residential	18	0	210	2450	23,250	279,167	3,513,333
Total suspended solid	0.0277	Industrial	9	1	<2.5	5.0	9.0	24.0	100.0
(mg/l)	<0.0001	Residential	18	0	3.0	8.8	16.5	29.3	307.0
BOD (mg/l)	0.0846	Industrial	9	0	2.5	7.3	9.6	25.6	39.6
	<0.0001	Residential	18	0	3.7	7.8	11.6	18.9	72.0
DO (mg/l)	0.9933	Industrial	9	0	4.6	6.7	8.8	10.1	11.7
	0.6842	Residential	18	0	3.5	5.4	6.6	7.7	9.6
Conductivity (mS/cm)	0.0025	Industrial	9	0	0.159	0.547	0.594	2.941	3.141
	0.0010	Residential	18	0	0.550	0.707	2.052	12.750	24.700
Salinity (ppt)	0.0007	Industrial	9	0	0.2	0.2	0.3	1.4	1.4
	0.0003	Residential	18	0	0.2	0.4	1.0	5.3	8.9
pH	0.1280	Industrial	9	0	5.93	6.99	8.58	8.87	9.14
	0.6778	Residential	18	0	6.87	7.18	7.39	7.63	8.24
Temperature (°C)	0.9268	Industrial	9	0	28.8	31.3	32.1	33.5	35.4
	0.0859	Residential	18	0	28.3	28.9	29.6	31.4	33.0

*^a^The Shapiro–Wilk normality test. P-values of less than 0.05 indicate non-normality. ^b^Summary statistics of data sets including non-detects were calculated with the non-parametric Kaplan–Meier method incorporating Efron bias correction. ^c^Data lower than the MLOQ.*

### MST Marker and Bacterial Monitoring

**FIGURE 2:**
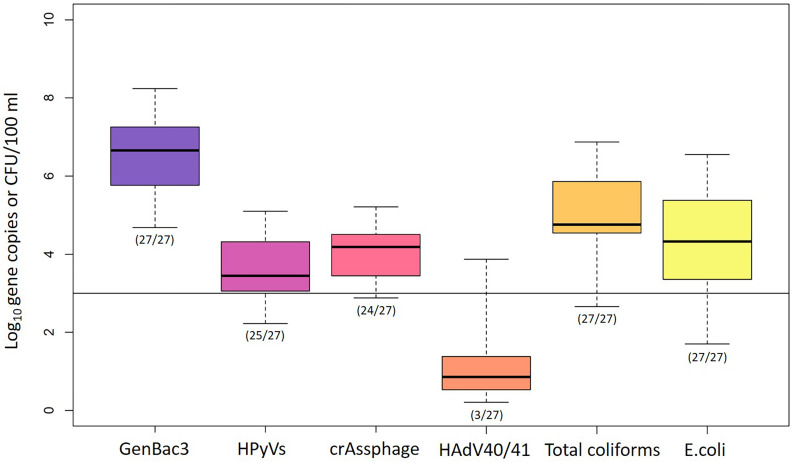
Abundance of microbial water quality parameters. Box plots represent the estimated 25th to 75th percentiles, with the median between. The whiskers exhibit the maximum and minimum values. The numbers in parentheses describe the number of positive samples/the number of total samples. A solid line indicates the highest MLOQ of 3.00 log_10_ copies/100 ml.

GenBac3 was detected in all samples with concentrations from 4.68 to 8.24 log_10_ copies/100 ml (Table 1 and Figure 2). HPyVs were detected in all but two samples (MLOQs of 2.10 and 2.40 log_10_ copies/100 ml), with a maximum of 5.10 log_10_ copies/100 ml, while crAssphage was in all but three samples (MLOQs of 2.40 and 3.00 log_10_ copies/100 ml) at concentrations up to 5.21 log_10_ copies/100 ml, as previously reported (Sangkaew et al., 2021). HAdV40/41 was found only in three residential sites from Event 1 at MP1 (2.82 log_10_ copies/100 ml), MP3 (2.59 log_10_ copies/100 ml), and MP10 (3.87 log_10_ copies/100 ml), while the rest were non-detects with an MLOQ of 2.40 log_10_ copies/100 ml. GenBac3 was found to be most abundant in the same samples, followed by crAssphage, HPyVs, and HAdV40/41, respectively (*p* < 0.05; paired Prentice–Wilcoxon test). Total coliforms and *E. coli* were positive in all samples, with levels from 453 to 7,333,333 and from 50 to 3,513,333 CFU/100 ml, respectively. A total of 85.19% of the samples exceeded the Thailand surface water quality standard of 20,000 MPN/100 ml (National Environment Board, 1994) when the CFU unit was comparable to the MPN unit (Gronewold and Wolpert, 2008). Moreover, no difference for microbial parameters was observed between sampling sites, indicating continual microbial contamination in the study area (Supplementary Table 7). CrAssphage and *E. coli* presented no temporal differences among the three events at each sampling site, while GenBac3, HPyVs, and total coliforms all represented significantly lower concentrations in Event 1 (*p* < 0.05; Supplementary Table 8).

### Correlation and Cluster Analyses

**FIGURE 3:**
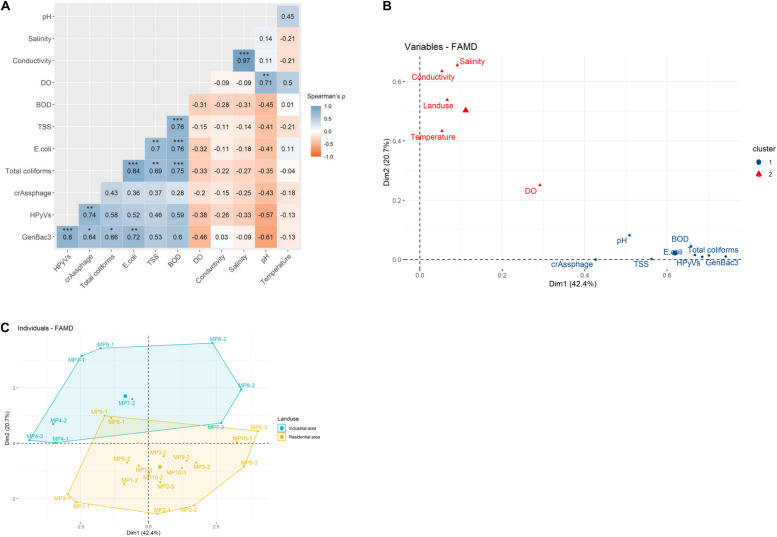
Multivariate analysis for quantitative correlation using Spearman’s rho on *U*-score rank with ^∗^, ^∗∗^, and ^∗∗∗^ indicating Holm’s adjusted *P*-value < 0.05, 0.01, and 0.001, respectively **(A)**, clustered principal component analysis (PCA) using factor analysis of mixed data (FAMD) for water quality parameters **(B)**, and clustered PCA using FAMD for water samples **(C)**.

Quantitative correlation analysis demonstrated a significantly strong correlation for the microbial parameters in pairs of GenBac3 and HPyVs, crAssphage, total coliforms, and *E. coli* (Spearman’s rhos = 0.64–0.80); a pair of HPyVs and crAssphage (rho = 0.74); and a pair of total coliforms and *E. coli* (rho = 0.84) (Figure 3A). Total coliforms and *E. coli* were also strongly correlated with TSS and BOD (rhos = 0.69–0.76). The physicochemical parameters were very strongly correlated between salinity and conductivity (rho = 0.97) and were strongly associated between pairs of BOD and TSS (rho = 0.76) and pH and DO (rho = 0.71).

A PCA was conducted, and the first two components displayed 42.4 and 20.7% of the variance (Supplementary Figure 2A). The variables contributing to the first two dimensions included GenBac3, salinity, total coliforms, BOD, HPyVs, conductivity, and *E. coli* (Supplementary Figure 2B). Two clusters of water quality parameters were demonstrated by the PCA (Figure 3B). Land use was more associated with physicochemical parameters in the first cluster (i.e., salinity, conductivity, water temperature, and DO). In contrast, the second cluster comprised physicochemical (i.e., pH, BOD, and TSS) and microbiological (i.e., GenBac3, HPyVs, total coliforms, *E. coli*, and crAssphage) variables (Figure 3B). When incorporating all the water quality parameters, the samples were clustered into two separate groups by land use type (Figure 3C). No significant differences in the abundance of MST and bacterial parameters between the two land use types were observed (Supplementary Table 9). DO and temperature were significantly higher and salinity was lower in industrial sites than in the residential sites (*p* < 0.05). At the same time, the rest of the physicochemical parameters provided no significant difference (Supplementary Table 9).

### QMRA and Sensitivity Analysis

The infection risks of HAdV40/41 were assessed using measured concentrations in the three monitoring campaigns. Due to positively detected HAdV40/41 in only three samples from the residential sites, we decided to increase the data set’s robustness by combining the data from all the sampling sites without segregating them into industrial and residential groups. The measured concentrations of HAdV40/41, crAssphage, and HPyVs were fitted to estimate the PDF in a log-normal distribution (Supplementary Table 10), and the predicted HAdV40/41 distribution was discerned from crAssphage and HPyVs. Due to higher exposure factors, the estimated Py increased from toilet flushing to aquaculture and food crop irrigation scenarios. The 95th percentiles of *P*_*y*_ predicted from crAssphage were 9.45 × 10^–3^, 6.34 × 10^–2^, and 2.63 × 10^–1^ pppy for toilet flushing, aquaculture, and agricultural irrigation, respectively (Table 2). In addition, the 95th percentiles of *P*_*y*_ predicted from HPyVs were 9.74 × 10^–3^, 6.48 × 10^–2^, and 2.70 × 10^–1^ pppy for toilet flushing, aquaculture, and irrigation, respectively (Table 2). The risk results from crAssphage and HPyVs were not significantly different due to a significant correlation between these two parameters. However, all the scenarios exceeded the US EPA risk benchmark at 10^–4^ pppy. Therefore, additional treatment was necessary. Based on the pooled detectable data in the dry season of the viruses, the requirements of additional treatment for HAdV40/41 to achieve an annual health risk of less than 10^–4^ pppy were estimated at 1.65–2.62, 2.48–3.46, and 3.15–4.13 log_10_ reduction (5th–95th percentiles) for toilet flushing, aquaculture, and food crop irrigation, respectively (Table 3). Various water treatment units were evaluated for each water reuse scenario (Supplementary Table 4). Chlorination was sufficient to achieve the 95th percentile (log_10_) reduction of HAdV40/41 for the toilet flushing reuse purpose. Moreover, the combination of depth filtration and chlorination was satisfactory to achieve the 95th percentile (log_10_) reduction of HAdV40/41 for aquaculture and food crop irrigation.

**TABLE 2:** Yearly infection probability (*P*_*y*_) of human adenovirus types 40 and 41 (HAdV40/41) predicted from crAssphage and HPyVs.

	Yearly infection probability (*P*_*y*_) in pppy					
Reuse purposes	5th percentile	25th percentile	Median	75th percentile	95th percentile	Mean
**Prediction from crAssphage**						
Toilet flushing	8.85 × 10^–4^	1.77 × 10^–3^	2.89 × 10^–3^	4.70 × 10^–3^	9.45 × 10^–3^	3.74 × 10^–3^
Aquaculture	5.94 × 10^–3^	1.20 × 10^–2^	2.51 × 10^–2^	3.14 × 10^–2^	6.34 × 10^–2^	2.51 × 10^–2^
Food crop irrigation	2.72 × 10^–2^	5.50 × 10^–2^	8.83 × 10^–2^	1.39 × 10^–1^	2.63 × 10^–1^	1.08 × 10^–1^
**Prediction from HPyVs**						
Toilet flushing	8.53 × 10^–4^	1.78 × 10^–3^	2.93 × 10^–3^	4.85 × 10^–3^	9.74 × 10^–3^	3.83 × 10^–3^
Aquaculture	5.80 × 10^–3^	1.21 × 10^–2^	1.99 × 10^–2^	3.23 × 10^–2^	6.48 × 10^–2^	2.56 × 10^–2^
Food crop irrigation	2.69 × 10^–2^	5.48 × 10^–2^	8.94 × 10^–2^	1.43 × 10^–1^	2.70 × 10^–1^	1.11 × 10^–1^

**TABLE 3:** Required log_10_ reduction of HAdV40/41 to achieve the US EPA annual infection risk benchmark of 10^–4^ per person per year as predicted by either crAssphage or HPyVs.

	Required log_10_ reduction			
	5th percentile	Median	95th percentile	Mean
Toilet flushing	1.65	2.13	2.62	2.14
Aquaculture	2.48	2.97	3.46	2.97
Food crop irrigation	3.15	3.63	4.13	3.64

**FIGURE 4:**
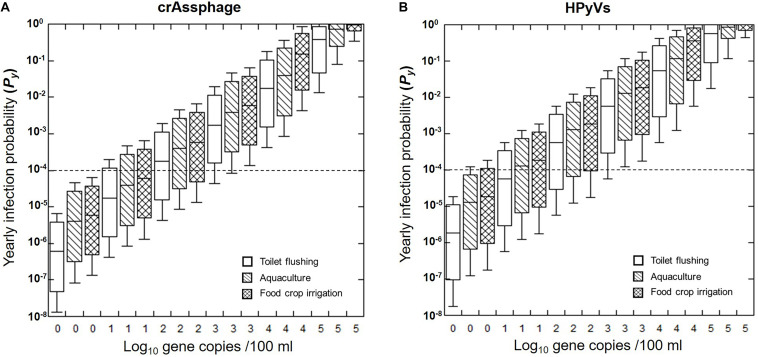
Predicted annual probability of infection (*Py*) for HAdV40/41 based on crAssphage **(A)** and HPyVs **(B)**. Box plots represent the estimated 25th to 75th percentiles, with the median between. The whiskers exhibit the 5th and 95th percentile values. The dashed lines indicate the US EPA annual risk benchmark of 10^–4^ pppy.

Furthermore, this study predicted the *P*_*y*_ from varying initial concentrations of crAssphage and HPyVs in the receiving water from 10^0^ to 10^5^ copies/100 ml (Figure 4). We found that the health risks expanded as the concentrations of markers increased. The 95th health risks of HAdV40/41 passed the US EPA annual infection risk benchmark of 10^–4^ pppy only in the following scenarios: (1) 10^0^ copies/100 ml of crAssphage for all three water reuse schemes, and (2) 10^0^ copies/100 ml of HPyVs for toilet flushing. Required log-removal can also be estimated from Figure 4. For instance, at the initial concentration of crAssphage at 10^4^ copies/100 ml, the receiving water requires approximately 3.2 log reduction treatment to achieve the US EPA benchmark at 10^–4^ for toilet flushing purposes. This is a useful tool for evaluating water quality and selecting the appropriate treatment for water reuse.

A sensitivity analysis was conducted to investigate the effects of each input variable on the estimated probability risks. The main contributor to risk variability in all the scenarios was the concentrations of HAdV40/41 in the receiving water (85.10 to 86.60%), which were similar when predicting HAdV40/41 from crAssphage and HPyVs (Supplementary Figure 3). Natural decay rates also had a reverse relationship with calculated risks (−12.30 to −13.50%). Less contributing factors included water use frequency per year, the volume of water, and the crAssphage or HPyV marker for prediction.

## Discussion

The MST marker abundance was investigated in surface water near the mixed land use of industrial and residential areas. Fecal contamination was indicated by GenBac3 level in this study (four to eight orders of magnitude in log_10_ copies/100 ml), which were slightly lower than those found in untreated sewage in Thailand (five to nine orders in log_10_ copies/100 ml) (Kongprajug et al., 2020), but slightly higher than the Tha Chin River in Central Thailand (three to seven orders in log_10_ copies/100 ml) (Kongprajug et al., 2019a). These GenBac3 levels were aligned with levels found in other geographical regions in urban rivers (five to seven orders), urban recreational beaches (two to five orders), and karst springs (four to seven orders) (Molina et al., 2014; Ohad et al., 2015; Devane et al., 2019). Human fecal contamination in this study was identified by the HPyVs and crAssphage markers of up to five orders in log_10_ copies/100 ml in this study, which were relatively lower than those presented in untreated sewage in Thailand (three to six and five to seven orders, respectively) (Kongprajug et al., 2019b; Sangkaew et al., 2021) and in the Tha Chin river (three to seven orders for crAssphage) (Kongprajug et al., 2019b), but slightly higher than in beach water in Thailand (up to four and three orders, respectively) (Sangkaew et al., 2021). The levels of HPyVs and crAssphage in Thailand’s freshwater were equivalent to those found in other geographical areas, as previously discussed (Sangkaew et al., 2021). Notably, marker abundance is also affected by the decay rate, which can be dependent on the water type (freshwater or marine water) and other environmental stressors (e.g., sunlight and indigenous microbiota) at different levels (Booncharoen et al., 2018; Korajkic et al., 2019).

The lack of spatial differences in the microbial indicators among the sampling sites indicated that the Nam Hu Canal passing through residential areas was affected by fecal contamination at levels similar to the Nam Cha and Lot Canals with upstream industrial sites (Figure 1). The Chak Mak Canal connects to the coast through a private land area, which disallowed sample collection at the downstream site. However, the Chak Mak Canal has been reported for its low flow and low current circulation in Pra Doo Bay (Singkran, 2012), leading to a minimal distribution of fecal pollution to the broader environment. This study emphasized the human fecal contamination impact on receiving water during dry weather, as has been previously reported (Sercu et al., 2009; Zimmer-Faust et al., 2018; Green et al., 2019; Gyawali et al., 2020). Sources of fecal contamination during dry weather could be from damaged sewer collection pipes, related infrastructure, or illicit discharges, which require further investigation. A total of 16,171 households are upstream of the Nam Cha and Lot Canals in the Huay Pong Sub-district, according to the 2019 National Population and Housing Census. The Chak Mak Canal runs through the Map Ta Phut Sub-district, which hosts 20,980 households, while the Nam Hu Canal passes by the Nean Phra Sub-district, which has 13,991 households. Stormwater runoff during rainfall events, even though not yet characterized in this study, potentially carries additional general or animal fecal microorganisms into the receiving water (Ahmed et al., 2019; Lee et al., 2020; Powers et al., 2020). However, the already-high level of fecal contamination in dry weather, as shown in this study, and especially the high percentage of samples exceeding the total coliform standards, has raised the attention for water quality mitigation strategies for expected higher pollution levels in the rainy season.

This study introduced a QMRA framework to promote a water reuse concept of receiving water in industrial communities. Adenoviruses have been widely used for QMRA in many applications, such as direct potable reuse (Amoueyan et al., 2019), indirect potable reuse to augment ground or surface water drinking sources (Amoueyan et al., 2019; Purnell et al., 2020), indirect wastewater reuse (Ahmed et al., 2018), biosolids for agricultural land applications (Hamilton et al., 2020), public drinking water supplies (Owens et al., 2020), natural recreational water (Federigi et al., 2019), stormwater runoff (Ahmed et al., 2019), and occupational exposure at wastewater treatment facilities (Carducci et al., 2018). In this study, HAdV40/41 distribution functions were estimated from crAssphage distributions, as supported by a significant correlation between these two DNA markers in wastewater (Farkas et al., 2019; Sirikanchana et al., 2020; Tandukar et al., 2020; Wu et al., 2020). Moreover, HPyVs and crAssphage have been reported for their significant correlation in wastewater and environmental waters (Sangkaew et al., 2021), thus supporting the fact that similar estimated risks of HAdV40/41 were observed in this study when predicted from either HPyVs or crAssphage. Human-specific MST markers, such as crAssphage (Crank et al., 2019) and HF183 (Boehm et al., 2015; Ahmed et al., 2018), have been used for QMRA; they provide the advantage of higher abundance than waterborne pathogens (Zhang et al., 2019).

In risk assessment, uncertainties are identified by a sensitivity analysis, and the uncertainties in this study were mainly due to HAdV40/41 abundance. With a relatively low positive percentage (11%) of HAdV40/41 DNA measurement, the simulated distribution functions from crAssphage and HPyVs prediction may have been confounded by different environmental conditions in each sampling event. Moreover, the annual risk overestimation could have occurred from substitution of MLOQ values for non-detects. Decreasing the MLOQ value would increase the possibility of detectable concentrations. Certain factors that could lower the detection limits include improved concentration recovery, larger processed water volume, and PCR inhibition alleviation (Kreader, 1996; Gibson et al., 2012; Petterson et al., 2015; Ahmed et al., 2020). Moreover, an assumption of infectious ratio values applied to the monitored DNA measurements (e.g., by molecular qPCR assays) contributes another uncertainty factor to the concentrations of infectious viruses to inform QMRA. Viral infectivity measurements, including cell culture and qPCR with azo-dye pretreatment methods (Ko et al., 2003; Leifels et al., 2019, 2021), could directly provide infectious concentrations of viruses for risk analysis. The differential decay characteristics of the HAdV40/41 and MST markers (i.e., crAssphage and HPyVs) in receiving water may also result in under- or over-estimation of the yearly infection risk (Greaves et al., 2020; McMinn et al., 2020). Therefore, future research is needed to investigate decay rates of the pathogens of interest in local environmental conditions and water matrices for accurate risk assessment. Studies to retrieve exposure factor values, including exposure volume and frequency, will also aid in precise assessment in local settings.

The US EPA drinking water annual risk benchmark of 10^–4^ pppy (US EPA, 1989) has been widely referenced in direct and indirect potable reuse (Amoueyan et al., 2019) and non-potable reuse (Hamilton et al., 2017; Simhon et al., 2020), although an attempt has been made to loosen the risk standard for irrigation purposes (Lim and Jiang, 2013). Moreover, the disability-adjusted life year (DALY) scores could be further calculated and emphasize the need for regional-specific information, including the total incidence, odds of severity, and illness duration (Hamilton et al., 2019). This study proposed three water reuse schemes: toilet flushing, aquaculture, and food crop irrigation. Toilet flushing reuse has been encouraged to promote the concept of eco-industrial parks (UNIDO, 2019). This study demonstrated that chlorination treatment is sufficient to decrease the viral risks of the receiving water and can promote water reuse practice in factories in the Map Ta Phut industrial estate. Chlorination is also a most common disinfection practice in Thailand due to its low cost and wide availability. Moreover, food crop irrigation and aquaculture, such as shellfish farming, are currently practiced in the local community. Although suggested pretreatments of depth filtration and chlorination may pose certain complications to implement, the infection risks calculated in this study have raised awareness of the current public health risks that will require further corrective actions. Additionally, effects of storm events have shown to be area-specific as higher abundance of human-specific fecal markers were observed after rainfall events in some studies (McGinnis et al., 2018; Wu et al., 2018), but not in others (García-Aljaro et al., 2017; Staley et al., 2018; Powers et al., 2020). Future wet-weather MST monitoring in this study area is therefore needed in order to evaluate risks and propose appropriate treatment units to reduce public health risks after storm events.

## Conclusion

Microbial fecal contamination from industrial and residential land uses in a coastal mixed-use area was investigated in this study using an integrated approach combining microbial water quality monitoring and the QMRA framework. Human fecal pollution as identified by sewage-specific MST markers was equally contributed by industrial and residential land uses. Annual infection risks for HAdV40/41 were assessed, and prior treatments were recommended before non-potable water reuse, including toilet flushing, aquaculture, and food crop irrigation. This first integrative microbial risk assessment approach in Thailand could enable effective preventive and mitigation measures for water quality management and water reuse in industrial and residential mixed land uses.

## Data Availability Statement

The original contributions generated for this study are included in the article/Supplementary Material, further inquiries can be directed to the corresponding author.

## Author Contributions

AK performed the experiments and analyzed data. NC conducted the experiments. TD carried out data and risk analyses. SM and KS conceived and designed the study. All authors discuss the results and wrote the manuscript.

## Conflict of Interest

The authors declare that the research was conducted in the absence of any commercial or financial relationships that could be construed as a potential conflict of interest.
