# Supplementary material for: Human Fecal Pollution Monitoring and Microbial Risk Assessment for Water Reuse Potential in a Coastal Industrial–Residential Mixed-Use Watershed
Source: Front Microbiol. 2021 Apr 20;12:647602. doi: 10.3389/fmicb.2021.647602 (PMC8093506; doi:10.3389/fmicb.2021.647602)
Supplement: Supplementary file 1 [file Data_Sheet_1.pdf]

Supplementary Material:

Human Fecal Pollution Monitoring and Microbial Risk Assessment for Water Reuse Potential in a Coastal Industrial-Residential Mixed-Use Watershed

Akechai Kongprajug[1,†], Thammanitchpol Denpetkul[2, †], Natcha Chyerochana[1], Skorn Mongkolsuk[1,3], and Kwanrawee Sirikanchana[1,3,*]

[1]*Research Laboratory of Biotechnology, Chulabhorn Research Institute, Bangkok, Thailand 10210;* [2] *Department of Social and Environmental Medicine, Faculty of Tropical Medicine, Mahidol University, Bangkok, Thailand 10400;* [3]*Center of Excellence on Environmental Health and Toxicology (EHT), Ministry of Education, Bangkok, Thailand 10400.*

[†]Contributed equally to this manuscript

*Correspondence: Kwanrawee Sirikanchana, kwanrawee@cri.or.th

```
  1     GATGCAACGC GAAGAACCTT ACCTGGCCTT GACATAGTAG AAACTTTCCA GAGATGGATT GGTGCCTTCG GGAAGAGAAA TTCCAAACGA ACTTGGAGAT
                                                                                     JTVFF
101     AGCTGGTTCT CTCCGAAATA GCTTTAGGGC TAGCCTCGGA ATTGAGAATG ATGGAGGTAG AGCACTGAAC TTTCTCTCTT AATAGACGCC CCACTTAATG
              JTVFP                                                                    JTVFR           SM2
201     CTGACACGGG CACTCTTCGC CTTCAAAGTG CTGCACCTCT TGGACTAGTG GACAAAACAC TAAAAGTTTT GTTTTCTAGC CCCCTAGTCT TTAGGGTCTT
                                                                          KGJ3
301     CTACCTTTCT TTTTTTCTTG GGTGGTGTTG AGTGTTGAGA ATCTGCTGTT GCTTCTTCAT CACTGGCAAA CATATCTTCA TGGCAAAATA AATCTTCATC
                                                          P6
401     CCATTTTTCA TTAAAGGAAC TCCACCAGGA CTCCCACTCT TCTGTTCCAT AGGTTGGCAC CGGTTTCCGC AGCTGGGGCA GTCGCAGGCG GCCACCGTGC

501     AGCCTTCGTC CCCCTAAGTT AAGACCAGGA CGGCTCGG
```

Supplementary Figure 1. Synthetic DNA standards (string 2) for HPyVs (forward primer – SM2; reverse primer – P6; and probe – KGJ3; McQuaig et al., 2009) and HAdV40/41 (forward primer – JTVFF; reverse primer – JTVFR; and probe – JTVFP; Ko et al., 2005)

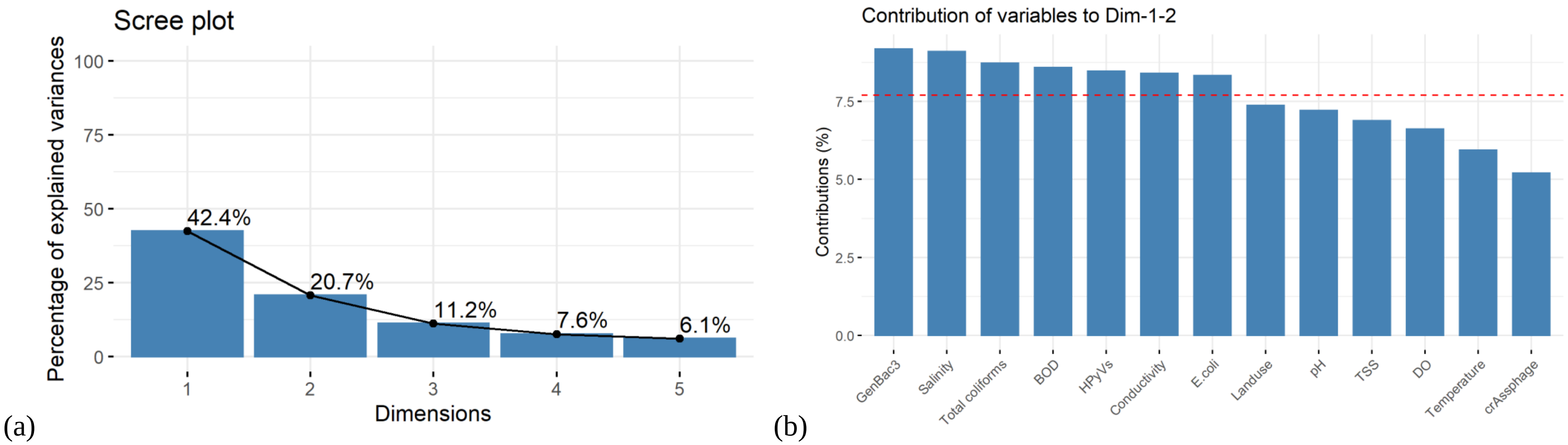

Supplementary Figure 2. PCA's scree plot (a) and contribution of water quality parameters (b). The red dashed line indicates the expected average contribution (=1/no. of variables).

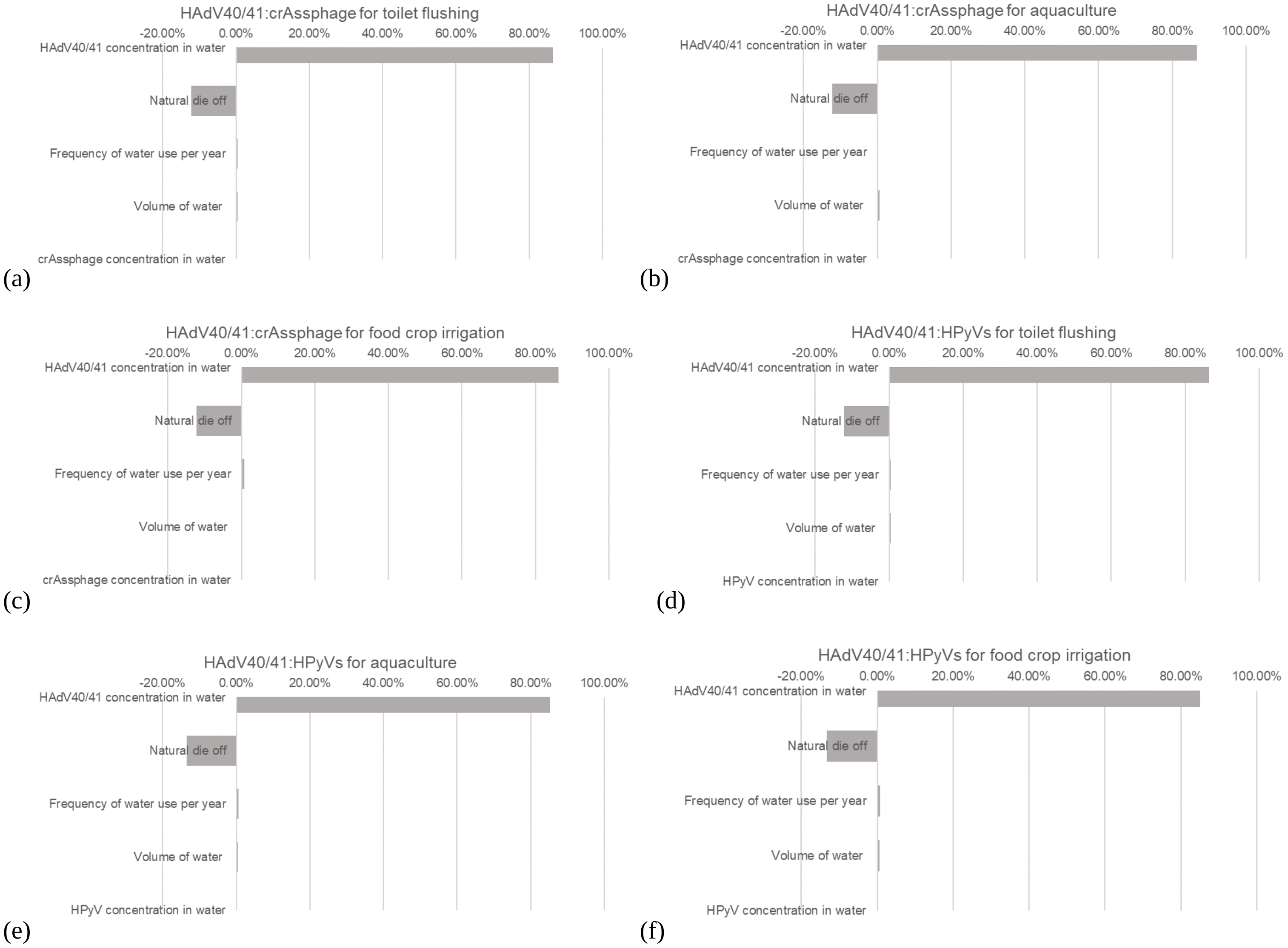

Supplementary Figure 3. Sensitivity analysis to evaluate contributions of each input variable to the annual probability risks (P_y) by predicting from crAssphage for toilet flushing (a), aquaculture (b), and food crop irrigation (c) and from HPyVs for toilet flushing (d), aquaculture (e), and food crop irrigation (f).

Supplementary Table 1. Locations of sampling sites

Sampling site	Latitude	Longitude
MP1	12.6702	101.1795
MP2	12.6786	101.1813
MP3	12.6780	101.1742
MP4	12.6915	101.1516
MP6	12.7163	101.1355
MP7	12.7378	101.1503
MP8	12.7463	101.1554
MP9	12.7189	101.1602
MP10	12.7094	101.1747

Supplementary Table 2. qPCR primers and hydrolysis probes used in this study

Assay name	Primer/probe name	Primer sequence (5' - 3')	Target microorganism	Target gene	Reference
GenBac3	GenBac3F	GGG-GTT-CTG-AGA-GGA-AGG-T	*Bacteroidetes*	16s rRNA	Siefring et al., 2008
	GenBac3R	CCG-TCA-TCC-TTC-ACG-CTA-CT			
	GenBacProbe	FAM-CAA-TAT-TCC-TCA-CTG-CTG-CCT-CCC-GTA-TAMRA			
HPyV	SM2	AGT-CTT-TAG-GGT-CTT-CTA-CCT-TT	human polyomaviruses JCV and BKV	T antigen	McQuaig et al., 2009
	P6	GGT-GCC-AAC-CTA-TGG-AAC-AG			
	KGJ3	FAM-TCA-TCA-CTG-GCA-AAC-AT-TAMRA			
crAssphage	CPQ_056F1	CAG-AAG-TAC-AAA-CTC-CTA-AAA-AAC-GTA-GAG	crAssphage	Genomic region 14731−14856	Stachler et al., 2017
	CPQ_056R1	GAT-GAC-CAA-TAA-ACA-AGC-CAT-TAG-C			
	CPQ_056P1	FAM-AAT-AAC-GAT-TTA-CGT-GAT-GTA-AC-TAMRA			
HAdV40/41	JTVFF	AAC-TTT-CTC-TCT-TAA-TAG-ACG-CC	Adenovirus Serotypes 40,41	Fiber gene	Ko et al., 2005
	JTVFR	AGG-GGG-CTA-GAA-AAC-AAA-A			
	JTVFP	FAM-CTG-ACA-CGG-GCA-CTC-TTC-GC-TAMRA			

Supplementary Table 3. Exposure scenarios and related factors

Reuse scenario	Route of exposure	Volume (ml)	Annual frequency	Reference
Toilet flushing	Inhalation (aerosol)	Uniform (0.009, 0.011)	Uniform (990, 1210)	NRMMC-EPHC-AHMC, 2006; Chhipi-Shrestha et al., 2017
Aquaculture	Ingestion	Uniform (2.7, 3.3)	Uniform (22.5, 27.5)	ANZECC and ARMCANZ, 2000; Chhipi-Shrestha et al., 2017
Food crop irrigation (lettuce)	Ingestion	Uniform (4.5, 5.5)[a]	Uniform (63,77)	NRMMC-EPHC-AHMC, 2006; Chhipi-Shrestha et al., 2017
Natural decay (log_{10} reduction)	-	Triangular (0.5, 0.5, 1)[b]	-	WHO, 2006; Chhipi-Shrestha et al., 2017

[a] Uniform distribution with minimum and maximum values
[b] Triangular distribution with minimum, peak, and maximum values

Supplementary Table 4. Log removal of treatment types for virus

Treatment	Virus $\log_{10}$ removal	Reference
Depth filtration	(1.95, 2.87, 3.61)[a]	Chhipi-Shrestha et al., 2017; Health Canada, 2019
Chlorination	(3.0, 3.5, 3.9)	Health Canada, 2010; Chhipi-Shrestha et al., 2017

[a] 50th, 75th, and 95th percentiles

Supplementary Table 5. Standard curve equations and qPCR assay characteristics

Assay name	$Slope_{std}$	Y-intercept	PCR efficiency[a]	ROQ[b]	ALOD[c]	ALOQ[d]	R^2
GenBac3[e]	-3.296	39.81	2.0109	5×10^6 - 50	20	50	0.999
HPyV[f]	-3.307	42.17	2.0063	5×10^6 - 50	40	50	0.998
crAssphage[g]	-3.277	41.38	2.0191	5×10^6 - 50	20	50	0.998
HAdV40/41	-3.587	39.23	1.9001	5×10^6 - 50	30	50	0.999

[a] PCR amplification efficiency (E_p) calculated by $E_p = 10^{(-1/Slope)}$

[b] Range of quantification (copies/reaction)

[c] Assay limit of detection (copies/reaction), determined as the lowest concentration that were detectable in all ten standard replicates

[d] Assay limit of quantification (copies/reaction), determined as the lowest concentration of the target gene that can be correctly quantified with a standard deviation of C_q <0.5

[e] From Kongprajug et al., 2019a

[f] From Sangkaew et al., 2021

[g] From Kongprajug et al., 2019b

Supplementary Table 6. Reproducibility of qPCR markers in field duplicates

Event	Sample	Log_{10} copies/100 ml			
		GenBac3	crAssphage	HPyVs	HAdV40/41
1	MP1.1/1	5.26	3.67	<MLOQ[a]	<MLOQ
	MP1.2/1	5.40	3.59	<MLOQ	<MLOQ
	Mean	5.33	3.63	NA[b]	NA
	SD[c]	0.07	0.04	NA	NA
	%CV[d]	1.24	1.06	NA	NA
2	MP1.1/2	6.75	4.37	3.56	<MLOQ
	MP1.2/2	6.55	4.26	3.03	<MLOQ
	Mean	6.65	4.31	3.29	NA
	SD	0.10	0.05	0.27	NA
	%CV	1.53	1.25	8.10	NA
3	MP1.1/3	7.11	3.41	2.85	<MLOQ
	MP1.2/3	7.06	3.56	2.72	<MLOQ
	Mean	7.09	3.49	2.78	NA
	SD	0.03	0.08	0.06	NA
	%CV	0.37	2.21	2.28	NA

[a] Method limit of quantification (log_{10} copies/100 ml), calculated from the assay limit of detection (ALOQ) by incorporating the sample's filtration volume and DNA extracted volume

[b] Not available

[c] Standard deviation

[d] Coefficient of variation

Supplementary Table 7. P-values of spatial comparison of microbial and physicochemical water quality parameters. Significant differences are indicated in bold by adjusted p-value below 0.0500.

Site comparison	GenBac3[a]	HPyVs[b]	crAssphage[b]	HAdV40/41[b]	Total coliforms[a]	*E.coli*[a]	TSS[b]	BOD[a]	DO[c]	Conductivity[c]	Salinity[c]	pH[c]	Temperature[c]
MP1 vs. MP2	1.0000	1.0000	1.0000	1.0000	1.0000	1.0000	1.0000	1.0000	0.6223	0.8978	0.9872	0.9869	0.9845
MP1 vs. MP3	1.0000	1.0000	1.0000	1.0000	1.0000	1.0000	1.0000	1.0000	0.9544	**0.0184**	**<0.0001**	0.9998	0.9629
MP1 vs. MP4	1.0000	1.0000	1.0000	1.0000	1.0000	1.0000	1.0000	1.0000	1.0000	**0.0033**	**<0.0001**	0.8355	1.0000
MP1 vs. MP6	1.0000	1.0000	1.0000	1.0000	1.0000	1.0000	1.0000	1.0000	0.8457	**0.0006**	**<0.0001**	0.9996	0.9986
MP1 vs. MP7	1.0000	1.0000	1.0000	1.0000	1.0000	1.0000	1.0000	1.0000	1.0000	**0.0006**	**<0.0001**	0.9884	1.0000
MP1 vs. MP8	1.0000	1.0000	1.0000	1.0000	1.0000	1.0000	1.0000	1.0000	0.9997	**0.0005**	**<0.0001**	1.0000	0.3943
MP1 vs. MP9	1.0000	1.0000	1.0000	1.0000	1.0000	1.0000	1.0000	1.0000	0.9997	**0.0006**	**<0.0001**	0.9217	0.9581
MP1 vs. MP10	1.0000	1.0000	1.0000	1.0000	1.0000	1.0000	1.0000	1.0000	0.9870	**0.0014**	**<0.0001**	0.9558	1.0000
MP2 vs. MP3	1.0000	1.0000	1.0000	1.0000	1.0000	1.0000	1.0000	1.0000	0.9974	0.2365	**0.0002**	1.0000	1.0000
MP2 vs. MP4	1.0000	1.0000	1.0000	NA	1.0000	1.0000	1.0000	1.0000	0.4529	0.0530	**0.0003**	0.3145	0.9054
MP2 vs. MP6	1.0000	1.0000	1.0000	NA	1.0000	1.0000	1.0000	1.0000	1.0000	**0.0096**	**<0.0001**	1.0000	1.0000
MP2 vs. MP7	1.0000	1.0000	1.0000	NA	1.0000	1.0000	1.0000	1.0000	0.8457	**0.0095**	**<0.0001**	1.0000	0.9790
MP2 vs. MP8	1.0000	1.0000	1.0000	NA	1.0000	1.0000	1.0000	1.0000	0.3254	**0.0088**	**<0.0001**	0.9478	0.0799
MP2 vs. MP9	1.0000	1.0000	1.0000	NA	1.0000	1.0000	1.0000	1.0000	0.8956	**0.0105**	**<0.0001**	1.0000	1.0000
MP2 vs. MP10	1.0000	1.0000	1.0000	1.0000	1.0000	1.0000	1.0000	1.0000	0.9852	**0.0225**	**<0.0001**	1.0000	0.9819
MP3 vs. MP4	1.0000	1.0000	1.0000	1.0000	1.0000	1.0000	1.0000	1.0000	0.8635	0.994	1.0000	0.5389	0.8438
MP3 vs. MP6	1.0000	1.0000	1.0000	1.0000	1.0000	1.0000	1.0000	1.0000	1.0000	0.7509	0.9792	1.0000	1.0000
MP3 vs. MP7	1.0000	1.0000	1.0000	1.0000	1.0000	1.0000	1.0000	1.0000	0.9969	0.749	0.9740	1.0000	0.9529
MP3 vs. MP8	1.0000	1.0000	1.0000	1.0000	1.0000	1.0000	1.0000	1.0000	0.7420	0.7289	0.9740	0.9962	0.0595
MP3 vs. MP9	1.0000	1.0000	1.0000	1.0000	1.0000	1.0000	1.0000	1.0000	0.9991	0.7757	0.9902	0.9966	1.0000
MP3 vs. MP10	1.0000	1.0000	1.0000	1.0000	1.0000	1.0000	1.0000	1.0000	1.0000	0.9297	1.0000	0.9991	0.9581
MP4 vs. MP6	1.0000	1.0000	1.0000	NA[d]	0.6474	1.0000	1.0000	1.0000	0.6953	0.9934	0.9223	0.5103	0.9751
MP4 vs. MP7	1.0000	1.0000	1.0000	NA	1.0000	1.0000	1.0000	1.0000	0.9982	0.9932	0.9099	0.3229	1.0000
MP4 vs. MP8	1.0000	1.0000	1.0000	NA	0.1682	1.0000	1.0000	0.6025	1.0000	0.9911	0.9099	0.9306	0.6106
MP4 vs. MP9	1.0000	1.0000	1.0000	NA	1.0000	1.0000	1.0000	0.9699	0.9947	0.9954	0.9529	0.1809	0.8324

MP4 vs. MP10	1.0000	1.0000	1.0000	NA	1.0000	1.0000	1.0000	1.0000	0.9400	1.0000	0.9992	0.2252	1.0000
MP6 vs. MP7	1.0000	1.0000	1.0000	NA	1.0000	1.0000	1.0000	1.0000	0.9697	1.0000	1.0000	1.0000	0.9977
MP6 vs. MP8	1.0000	1.0000	1.0000	NA	1.0000	1.0000	1.0000	1.0000	0.5484	1.0000	1.0000	0.9943	0.1368
MP6 vs. MP9	1.0000	1.0000	1.0000	NA	1.0000	1.0000	1.0000	1.0000	0.9852	1.0000	1.0000	0.9978	0.9999
MP6 vs. MP10	1.0000	1.0000	1.0000	NA	1.0000	1.0000	1.0000	1.0000	0.9997	1.0000	0.9987	0.9995	0.9982
MP7 vs. MP8	1.0000	1.0000	1.0000	NA	1.0000	1.0000	1.0000	1.0000	0.9870	1.0000	1.0000	0.9519	0.4211
MP7 vs. MP9	1.0000	1.0000	1.0000	NA	1.0000	1.0000	1.0000	1.0000	1.0000	1.0000	1.0000	1.0000	0.9472
MP7 vs. MP10	1.0000	1.0000	1.0000	NA	1.0000	1.0000	1.0000	1.0000	0.9997	1.0000	0.9981	1.0000	1.0000
MP8 vs. MP9	1.0000	1.0000	1.0000	NA	1.0000	1.0000	1.0000	1.0000	0.9728	1.0000	1.0000	0.8213	0.0567
MP8 vs. MP10	1.0000	1.0000	1.0000	NA	1.0000	1.0000	1.0000	1.0000	0.8548	1.0000	0.9981	0.8796	0.4076
MP9 vs. MP10	1.0000	1.0000	1.0000	NA	1.0000	1.0000	1.0000	1.0000	1.0000	1.0000	0.9997	1.0000	0.9529

[a] Kruskal-Wallis test with Dunn's multiple comparisons test for non-normal data
[b] Generalized Wilcoxon test with Holm’s bias correction for data sets containing non-detects
[c] One-way ANOVA with Tukey's multiple comparisons test for normal data
[d] Not available

Supplementary Table 8. P-values of temporal comparison of microbial and physicochemical water quality parameters. Significant differences are indicated in bold by adjusted p-value below 0.0500.

Parameter	Two-tailed test			One-tailed test					
	Event 1 ≠ 2	Event 1 ≠ 3	Event 2 ≠ 3	Event 1<2	Event 1>2	Event 1<3	Event 1>3	Event 2<3	Event 2>3
GenBac3[a]	**0.0141**	**0.0158**	0.6918	**0.0070**	0.9930	**0.0079**	0.9921	-	-
HPyVs[b]	**0.0112**	0.1851	0.8702	**0.0091**	0.9909	-	-	-	-
crAssphage[b]	0.6460	0.8909	0.6260	-	-	-	-	-	-
HAdV40/41[b]	0.0845	0.0845	NA[d]	-	-	-	-	-	-
Total coliforms[c]	0.4258	**0.0273**	0.0977	-	-	**0.0136**	0.9902	-	-
E.coli[c]	0.2500	0.0547	0.1289	-	-	-	-	-	-
TSS[b]	0.5963	0.0540	**0.0171**	-	-	-	-	**0.0086**	0.9915
BOD[c]	0.4961	**0.0391**	0.1289	-	-	**0.0195**	0.9863	-	-
DO[a]	0.0775	**0.0420**	0.8038	-	-	0.9790	**0.0210**	-	-
Conductivity[c]	1.0000	0.7344	0.1641	-	-	-	-	-	-
Salinity[c]	0.6750	0.5513	0.2345	-	-	-	-	-	-
pH[c]	**0.0273**	**0.0117**	0.9442	0.9902	**0.0137**	0.9961	**0.0059**	-	-
Temperature[a]	0.9285	0.4947	0.3205	-	-	-	-	-	-

[a] Paired t-test for normal data
[b] Paired Prentice–Wilcoxon test for data sets containing non-detects
[c] Wilcoxon signed-rank test for non-normal data
[d] Not available

Supplementary Table 9. Statistical significant differences of microbial and physicochemical parameters between industrial and residential land uses. Significant differences are indicated in bold by adjusted p-values below 0.0500.

Parameter	Two tailed test	One-tailed test	
	Industrial ≠ Residential	Industrial < Residential	Industrial > Residential
GenBac3[a]	0.4242		
HPyVs[b]	0.6619		
crAssphage[b]	0.2166		
HAdV40/41[b]	0.2033		
Total coliforms[c]	0.2317		
E.coli[c]	0.6314		
TSS[b]	0.4100		
BOD[c]	0.7912		
DO[a]	**0.0270**	0.9778	**0.0222**
Conductivity[c]	0.0525		
Salinity[c]	**0.0307**	**0.0071**	0.9929
pH[a]	0.0554		
Temperature[a]	**0.0017**	0.9965	**0.0035**

[a] t-test for normal data
[b] Generalized Wilcoxon test for data sets containing non-detects
[c] Mann Whitney test for non-normal data

Supplementary Table 10. Probability density functions (PDF) of the virus concentrations

Element	Number of samples	PDF type	Estimation parameters (gene copies/100 ml)
Estimated crAssphage	27	Log normal	Location = 0, Mean 45858.99, SD[a] = 204585.35
Estimated HPyVs	27	Log normal	Location = 112.56, Mean 40277.55, SD = 475301.72
Estimated HAdV40/41	27	Log normal	Location = 0, Mean 374.58, SD = 283.50

[a]Standard deviation

References

ANZECC, and ARMCANZ (2000). Australian and New Zealand guidelines for fresh and marine water quality. Volume 1. The Guidelines. *Natl. Water Qual. Manag. Strateg.* 1, 314. Available at: http://www.dofa.gov.au/infoaccess/.

Chhipi-Shrestha, G., Hewage, K., and Sadiq, R. (2017). Microbial quality of reclaimed water for urban reuses: Probabilistic risk-based investigation and recommendations. *Sci. Total Environ.* 576, 738–751. doi:10.1016/j.scitotenv.2016.10.105.

Health Canada (2010). *Canadian Guidelines for Domestic Reclaimed Water for Use in Toilet and Urinal Flushing*.

Health Canada (2019). *Guidelines for Canadian drinking water quality: guideline technical document - arsenic*. Available at: http://www.hc-sc.gc.ca/ewh-semt/alt_formats/hecs-sesc/pdf/pubs/water-eau/sum_guide-res_recom/summary-sommaire-eng.pdf.

Ko, G., Jothikumar, N., Hill, V. R., and Sobsey, M. D. (2005). Rapid detection of infectious adenoviruses by mRNA real-time RT-PCR. *J. Virol. Methods* 127, 148–153. doi:10.1016/j.jviromet.2005.02.017.

Kongprajug, A., Chyerochana, N., Somnark, P., Kampaengthong, P. L., Mongkolsuk, S., and Sirikanchana, K. (2019a). Human and animal microbial source tracking in a tropical river with multiple land use activities. *Int. J. Hyg. Environ. Health* 222, 645–654. doi:10.1016/j.ijheh.2019.01.005.

Kongprajug, A., Mongkolsuk, S., and Sirikanchana, K. (2019b). CrAssphage as a potential human sewage marker for microbial source tracking in Southeast Asia. *Environ. Sci. Technol. Lett.* 6, 159–164. doi:10.1021/acs.estlett.9b00041.

McQuaig, S. M., Scott, T. M., Lukasik, J. O., Paul, J. H., and Harwood, V. J. (2009). Quantification of human polyomaviruses JC virus and BK virus by TaqMan quantitative PCR and comparison to other water quality indicators in water and fecal Samples. *Appl. Environ. Microbiol.* 75, 3379–3388. doi:10.1128/AEM.02302-08.

NRMMC-EPHC-AHMC (2006). Australia Guidelines for Water Recycling: Managing Health and Environmental Risks (Phase 1). *Natl. Water Qual. Manag. Strateg.*, 415.

Sangkaew, W., Kongprajug, A., Chyerochana, N., Ahmed, W., Rattanakul, S., Denpetkul, T., et al. (2021). Performance of viral and bacterial genetic markers for sewage pollution tracking in tropical Thailand. *Water Res.* 190, 116706. doi:https://doi.org/10.1016/j.watres.2020.116706 WR 116706.

Siefring, S., Varma, M., Atikovic, E., Wymer, L., and Haugland, R. A. (2008). Improved real-time PCR assays for the detection of fecal indicator bacteria in surface waters with different instrument and reagent systems. *J. Water Health* 6, 225–237. doi:10.2166/wh.2008.022.

Stachler, E., Kelty, C., Sivaganesan, M., Li, X., Bibby, K., and Shanks, O. C. (2017). Quantitative crAssphage PCR assays for human fecal pollution measurement. *Environ. Sci. Technol.* 51, 9146–9154. doi:10.1021/acs.est.7b02703.

WHO (2006). *WHO Guidelines for the Safe Use of Wastewater, Excreta and Greywater. Volume 2 Wastewater Use in Agriculture*. Available at: http://whqlibdoc.who.int/publications/2006/9241546832_eng.pdf.
